# Improving Plasma‐Catalytic Ammonia Synthesis Using a Coaxial Double‐Helix‐Electrode Reactor

**DOI:** 10.1002/cssc.202502695

**Published:** 2026-03-29

**Authors:** Shijie Xian, Xiaolan Fu, Shaowei Chen, Liping Cao, Tianqi Liu, Yibing Mu, Xiaolei Fan, Jiangqi Niu

**Affiliations:** ^1^ College of Chemistry and Materials Engineering Wenzhou University Wenzhou China; ^2^ Wenzhou Key Laboratory of Novel Optoelectronic and Nano Materials Institute of Wenzhou Zhejiang University Wenzhou China; ^3^ Department of Materials Science and Engineering Zhejiang University Hangzhou China; ^4^ State Key Laboratory of Materials‐Oriented Chemical Engineering College of Chemical Engineering Nanjing Tech University Nanjing China; ^5^ Ningbo China Beacons of Excellence Research and Innovation Institute University of Nottingham Ningbo China Ningbo China; ^6^ Department of Chemical Engineering School of Engineering The University of Manchester Manchester UK; ^7^ Department of Chemical and Biomolecular Engineering National University of Singapore Singapore

**Keywords:** ammonia synthesis, dielectric barrier discharge (DBD), electric‐field simulation, plasma catalysis, reactor design

## Abstract

Developing energy‐efficient ammonia synthesis under mild and carbon‐neutral conditions remains a major challenge for sustainable nitrogen fixation. Here, we present a coaxial double‐helix‐electrode‐based double‐dielectric barrier discharge (DBD) reactor, termed a “double‐helix” design, featuring dual quartz barriers and symmetric high‐voltage and grounded electrodes to achieve uniform, high‐intensity volume discharge for plasma‐catalytic ammonia synthesis. Three‐dimensional electrostatic simulations demonstrate that this configuration generates a strongly coupled and spatially homogeneous electric field (∼7 × 10^6^ V m^−1^), significantly outperforming conventional single‐dielectric DBD designs (∼1 × 10^6^ V m^−1^). An optimized Ni electrode with a 1 mm winding pitch increases electron density, as evidenced by optical emission spectroscopy (OES, *I*_N2+_(425 nm)/*I*_N2*_(335 nm) = 0.15). Under plasma‐only operation, the double‐helix DBD reactor produces approximately 2.5‐fold higher NH_3_ concentration than a conventional DBD at identical power input. When integrated with a Ni/Al_2_O_3_ catalyst, synergistic plasma‐catalyst interactions further enhance ammonia yield and energy efficiency, achieving an energy yield of up to 3.68 g NH_3_ kWh^−1^ under 5.92 W discharge. Comprehensive analysis combining electric‐field simulations, transient discharge imaging, and catalytic performance measurements elucidates the intrinsic coupling between electrode architecture, discharge physics, and catalytic function. This work demonstrates that electric‐field engineering is an effective strategy for enabling stable volume discharge and enhancing plasma‐catalytic ammonia synthesis, offering a generic design principle for next‐generation low‐carbon nitrogen‐fixation systems.

## Graphical abstract

A symmetric double‐dielectric, double‐helix dielectric barrier discharge (DBD) reactor enables uniform volume discharge, enhanced energetic electrons, and strong plasma‐catalyst synergy, delivering high‐efficiency ammonia synthesis under mild, low‐carbon conditions.

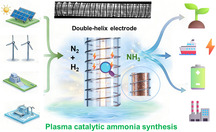

## 1 Introduction

Ammonia is one of the most indispensable chemical feedstocks in modern industry, with critical roles in fertilizer production, refrigeration, pharmaceuticals, and emerging hydrogen energy storage technologies [1]. In the context of the global transition toward decarbonized energy systems, ammonia has attracted renewed interest as a carbon‐neutral fuel and hydrogen carrier due to its high hydrogen density, ease of storage and transport, and favorable combustion characteristics [2]. At present, industrial ammonia synthesis is dominated by the Haber–Bosch process [3], a mature technology but one that requires severe operating conditions (400–500°C, 15–30 MPa), substantial capital investment, and a continuous supply of fossil fuel‐derived hydrogen. These factors collectively result in considerable CO_2_ emissions, underscoring the urgent need for green ammonia synthesis routes capable of operating under mild conditions and compatible with intermittent renewable energy sources.

Nonthermal plasma technology has emerged as a promising alternative for sustainable ammonia production [4], owing to its ability to activate N_2_ and H_2_ molecules at near‐ambient temperatures and pressures. Through inelastic collisions between energetic electrons and gas molecules, plasma enables the excitation, vibrational activation, and partial dissociation of the exceptionally stable triple bond in N_2_, providing an unconventional reaction pathway toward low‐carbon, distributed ammonia synthesis [5]. Current research in this field centers on two primary directions: (i) the development of plasma‐catalyst systems [6] that incorporate transition metals, metal oxides, or composite materials to enhance nitrogen activation and ammonia selectivity, and (ii) the structural optimization of plasma reactors [7] to improve discharge stability, electric‐field uniformity, and energy efficiency. However, compared with the extensive efforts devoted to catalyst engineering, systematic investigations of plasma reactor architecture and electric‐field design remain relatively limited.

Dielectric barrier discharge (DBD) reactors constitute the most widely explored configuration for plasma‐driven ammonia synthesis [8]. A conventional DBD reactor typically adopts a coaxial structure comprising an inner electrode, an outer electrode, and an intervening dielectric barrier, commonly quartz or glass [4]. The inner electrode is often a metallic rod or wire (e.g., copper [9], stainless steel [10], or tungsten [11]), whereas the outer electrode is formed by a metallic mesh, wire, or foil wrapped around the dielectric surface [12]. The dielectric barrier functions to limit current, prevent arcing, and sustain nonthermal plasma conditions. Although multiple variations of DBD architectures have been developed (including packed‐bed [13], layered [14], and multielectrode [15] configurations), challenges remain in achieving uniform discharge, sufficiently strong electric fields, and efficient plasma‐catalyst coupling. In principle, helix electrodes can significantly improve electric‐field uniformity by redistributing the local field along the discharge path; however, this geometry has received little attention in the plasma‐catalysis community. These limitations continue to restrict energy efficiency and ammonia production rates [16].

To address these challenges, we propose that a symmetric dual‐dielectric, double‐helix‐electrode configuration can fundamentally reshape the spatial electric‐field distribution, thereby enhancing discharge uniformity, elevating electron density, and ultimately overcoming performance bottlenecks in plasma‐driven ammonia synthesis. Guided by this hypothesis, we designed and fabricated a novel coaxial plasma reactor featuring a double‐quartz‐tube geometry. Unlike previously reported single‐helix DBD reactors [17, 18], in which only one helical electrode is employed and the resulting electric field is inherently single‐sided, the present design incorporates two helical electrodes arranged symmetrically on the inner and outer dielectric tubes. Although dual‐dielectric DBD reactors have also been reported, these systems typically retain asymmetric electrode configurations and therefore do not establish symmetric electric field coupling across the discharge gap. In contrast, the proposed double‐helix architecture enables mirror‐symmetric, dual‐sided electric‐field coupling, producing a more uniform field distribution and an enlarged effective discharge region.

Structurally, the reactor consists of two concentric quartz tubes. The inner tube is packed with copper powder and wound with a metal wire serving as the high‐voltage electrode, and the outer tube is wrapped with a copper wire functioning as the ground electrode. The discharge primarily occurs in the annular region between the two dielectric layers. Owing to the presence of dual dielectric barriers and a symmetric electrode arrangement, the reactor generates a strongly coupled and spatially homogeneous electric field. The intensified local electric field near the helical inner electrode promotes the formation of predischarges by accelerating seed electrons to energies sufficient for impact ionization. These predischarges act as localized electron reservoirs that enrich the discharge gap with seed electrons and metastable species, thereby reducing the effective breakdown voltage and facilitating the rapid development of Townsend avalanches. Consequently, the reactor exhibits improved plasma ignition behavior, enhanced discharge continuity, and more extensive volume discharges. This preionization effect ultimately strengthens plasma‐catalyst interactions, increases discharge stability, and improves energy utilization efficiency.

This study systematically investigates the interplay between reactor architecture, discharge characteristics, and ammonia synthesis performance in this newly developed double‐helix‐electrode‐structured double‐dielectric DBD reactor (hereafter denoted as the double‐helix DBD). The insights gained provide an experimental foundation and design principles for next‐generation plasma reactors aimed at high‐efficiency, low‐carbon ammonia production within the emerging nitrogen circular economy.

## 2 Experimental

### 2.1 Catalyst Preparation and Characterization

#### 2.1.1 Chemicals and Materials

Nickel (II) nitrate hexahydrate (Ni(NO_3_)_2_ · 6H_2_O), nanosized zirconia (ZrO_2_, 99.99%, particle size <100 nm), and nanosized alumina (Al_2_O_3_, 99.99%, average particle size of ∼10 nm) were purchased from Aladdin Industrial Corporation (Shanghai Aladdin Biochemical Technology Co., Ltd, China). All chemicals were of analytical grade and used as received without further purification.

#### 2.1.2 Catalyst Preparation

Ni/Al_2_O_3_ and Ni/ZrO_2_ catalysts with a nominal Ni loading of 3 wt% were prepared by the incipient wetness impregnation method. For Ni/Al_2_O_3_ [19], 1 g of Al_2_O_3_ support was dispersed in 25 mL of deionized water, followed by the addition of 0.131 g of Ni(NO_3_)_2_ · 6H_2_O dissolved in 25 mL of deionized water. The suspension was stirred at room temperature for 4 h and then evaporated to dryness in a water bath at 80°C. The resulting solid was dried overnight at 60°C, calcined in a muffle furnace at 450°C for 2 h under static air (heating rate: 5°C min^−1^), and subsequently reduced in a tube furnace at 400°C for 2 h under flowing H_2_ (5 mL min^−1^). After cooling to room temperature under an inert atmosphere, the Ni/Al_2_O_3_ (3 wt%) catalyst was obtained. Ni/ZrO_2_ (3 wt%) was prepared following the same procedure, substituting Al_2_O_3_ with an equivalent mass of ZrO_2_. All catalysts were stored in a vacuum desiccator prior to use to avoid moisture uptake.

#### 2.1.3 Catalyst Characterization

The catalysts were comprehensively characterized to evaluate their structural, surface, and textural properties using X‐ray diffraction (XRD), temperature‐programmed techniques, nitrogen adsorption porosimetry, and transmission electron microscopy (TEM). XRD patterns were collected on a SmartLab diffractometer (Rigaku Corporation, Japan) to identify the crystalline phases present. Temperature‐programmed reduction and desorption measurements (H_2_‐TPR, H_2_‐TPD, NH_3_‐TPD, and N_2_‐TPD) were performed using a TP‐5080 analyzer (Xianquan Scientific Instruments Co., Ltd, China). Prior to each measurement, the samples were pretreated under flowing Ar (30 mL min^−1^) by heating from room temperature to 400°C at a rate of 10°C min^−1^, followed by cooling to 20°C. The samples were then saturated with the corresponding probe gas (10 vol% H_2_/Ar for H_2_‐TPR and H_2_‐TPD, NH_3_ for NH_3_‐TPD, and N_2_ for N_2_‐TPD), after which temperature‐programmed heating was conducted to record reduction or desorption profiles. Textural properties were determined by N_2_ adsorption–desorption measurements using an ASAP 2460 system (Micromeritics Instrument Corporation, USA). TEM images were acquired on a Talos F200S microscope (Thermo Fisher Scientific, USA) operated at 200 kV to examine the dispersion and morphology of Ni species on the supports. Prior to analysis, the samples were ultrasonically dispersed in ethanol and deposited onto carbon‐coated copper grids.

### 2.2 Reactor Configurations and Experimental Rig

**FIGURE 1:**
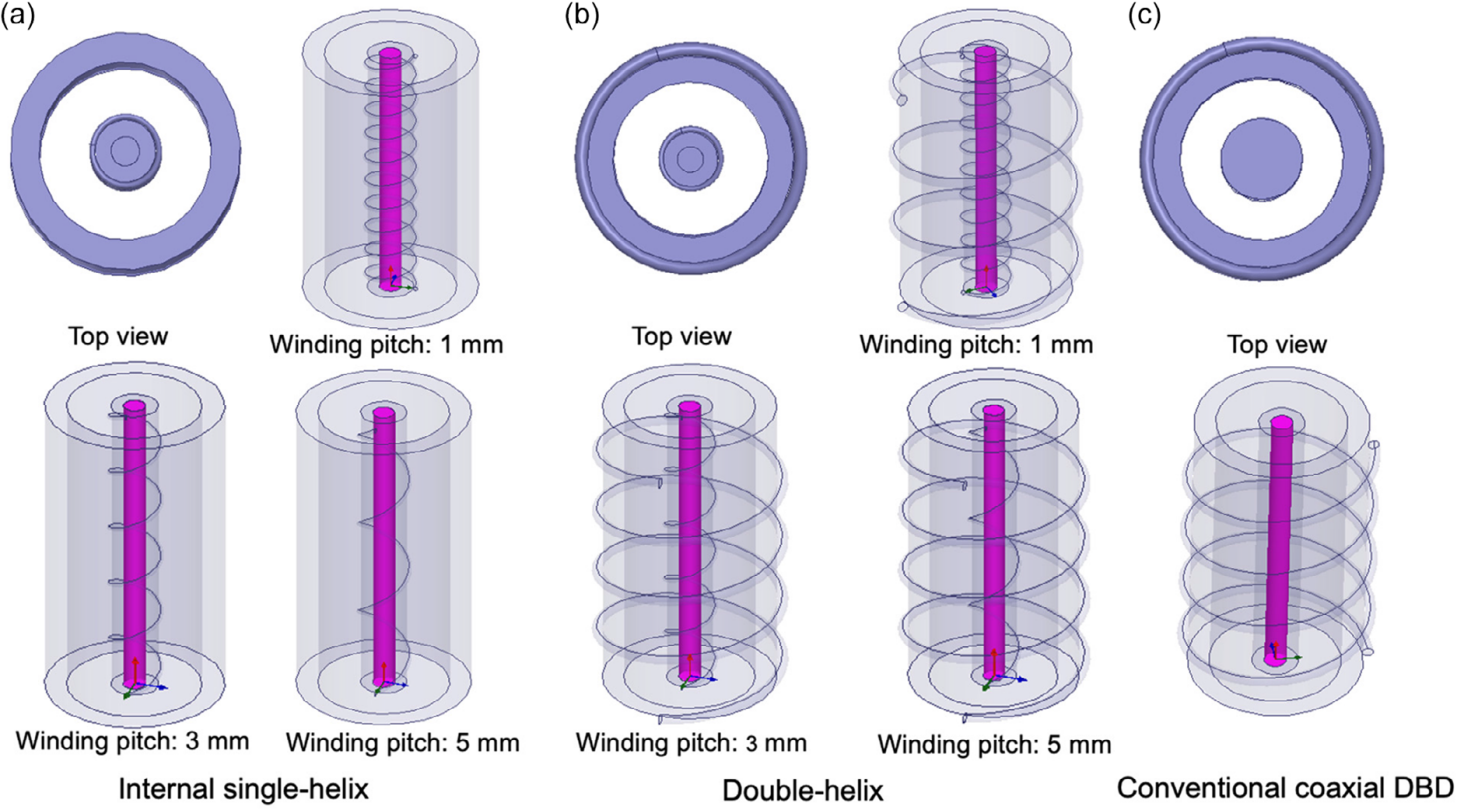
Models of three discharge configurations for the simulation study. (a) Internal single‐helix structure with winding pitches of 1, 3, and 5 mm; (b) double‐helix structure with winding pitches of 1, 3, and 5 mm; and (c) conventional coaxial DBD simulation structure schematic.

Three DBD reactors with coaxial double‐quartz‐tube structures were designed to evaluate the effect of discharge architecture on plasma‐assisted ammonia synthesis: (i) the internal single‐helix electrode design (Figure 1a), (ii) the coaxial double‐helix electrode design (Figure 1b), and (iii) a conventional coaxial configuration (Figure 1c). All reactors had identical geometrical dimensions. Each consisted of two concentric quartz tubes: an inner tube (I.D. = 1 mm, O.D. = 3 mm) and an outer tube (I.D. = 6 mm, O.D. = 8 mm). The inner tube was filled with copper powder to ensure stable electrical contact. High‐voltage excitation was supplied by a 0.2 mm metal wire (Cu, Ni, or W) helically wound around the inner tube over a length of 7 cm. When required, the grounded electrode consisted of a 0.5 mm copper wire wound around the outer tube at a 3 mm pitch. The discharge zone was defined by the annular gap between the two quartz tubes.

In the internal single‐helix structure, a helical metal wire was placed on inner quartz tube as high‐voltage electrode, while the inner quartz tube was filled with copper powder to serve as the ground electrode, producing a single‐sided discharge field. In the double‐helix structure, based on the internal single‐helix configuration, an outer quartz tube wrapped with a copper wire serving as the ground electrode was added, creating a symmetric dual‐sided discharge that enhanced electric‐field uniformity. In the conventional coaxial DBD, the helix electrode was placed on the outer tube, while the copper‐filled inner tube served as the high‐voltage electrode, confining the discharge to the annulus.

The experimental rig (Figure S2) employed a pulsed AC power supply (CTP‐2000K, Nanjing Suman Plasma Technology Co., China) that generated discharges at 5–8 kV and 7 kHz. Voltage and current were monitored using a high‐voltage probe and a Rogowski coil. Discharge power was determined from Lissajous figures using an external 0.22 µF capacitor (*Q* = *CV*, where *Q* is the charge stored on the external capacitor (C); *C* is the capacitance of the external capacitor (F); and *V* is the voltage across the capacitor measured by the probe (V)). Optical emission spectra (OES) were acquired using an AvaSpec‐ULS4096CL‐EVO spectrometer (200–1100 nm, 0.4 nm resolution, Avantes BV, The Netherlands). The emission intensity ratio *R* = *I*_N2+_(425 nm)/*I*_N2*_(335 nm) was used as an indicator of relative electron excitation energy. High‐speed imaging (exposure: 0.5–16 ms; SPL‐PLSMA‐IMV3, Hangzhou SPL Photonics Co. Ltd, China) was used to visualize microdischarge behavior and assess temporal–spatial discharge uniformity.

### 2.3 Plasma‐Assisted Ammonia Synthesis

#### 2.3.1 Plasma‐Only Experiments

Plasma‐only tests were conducted to evaluate the influence of reactor geometry and electrode parameters on ammonia formation. Total gas flow rates were calibrated using the soap‐bubble method [20]. A premixed N_2_/H_2_ (1:1) gas stream was supplied at 40 mL min^−1^. For each operating condition, discharge characteristics, including Lissajous figures, current–voltage waveforms, and OES spectra, were collected prior to ammonia quantification.

Outlet gases were analyzed online using a gas chromatograph (GC‐LTZ, Fuli Instruments, China). The GC was calibrated using an external standard method. A certified NH_3_ calibration gas (100 ppm in N_2_) was used as the primary standard. Serial dilutions (10–100 ppm) were prepared by mixing the standard gas with high‐purity N_2_ through a mass‐flow‐controlled dilution manifold. For each concentration level, the GC peak area was measured three times, and a linear calibration curve (peak area vs. NH_3_ concentration) was obtained (*R* ^2^ > 0.999, Figure S3). For each condition, three consecutive GC injections were performed at 10 min intervals under steady discharge operation, and the reported NH_3_ concentration represents the average of the three measurements. Ammonia quantification employed external standard calibration prior to each experiment.

The specific energy input (SEI) and ammonia energy yield (*E*) were calculated using Equations (1)–(2):

(1) $$\mathrm{SEI}=\frac{\text{discharge power }\left(W\right)}{Q_{\mathrm{gas}}}$$

(2) $$E_{\mathrm{NH}3}=\frac{{\text{moles of NH}}_{3}\text{yield}\;(\text{mol}s^{-1})\times 17}{\text{discharge power }\left(\mathrm{kW}\right)}$$

where *Q*_gas_ is the inlet flow rate.

For both the conventional DBD and the double‐helix configurations, comparative experiments were conducted across nine reactor‐electrode combinations, employing Cu, Ni, or W high‐voltage wires with winding pitches of 1, 3, and 5 mm. Winding pitches smaller than 1 mm were not investigated due to geometric constraints imposed by the interelectrode gap, which would hinder effective catalyst packing in subsequent plasma‐catalytic experiments. Discharge power was varied at 13, 17, and 21 W.

#### 2.3.2 Plasma‐Catalyst Experiments

Catalyst powders were pelletized and sieved to 425–600 μm to ensure compatibility with the 1 mm interelectrode spacing while maintaining stable packed‐bed operation. This standardized particle size was applied consistently throughout the study to enable direct comparison across different plasma configurations. The catalysts were pretreated in situ under an Ar/H_2_ (3:2) mixture (50 mL min^−1^) at 7.4 kV and 7 kHz for 15 min. Following identification of the optimal plasma configuration (Ni electrode, 1 mm pitch), 0.2 g of catalyst was thoroughly mixed with 0.2 g of quartz sand and loaded into the discharge zone. Ni/Al_2_O_3_ and Ni/ZrO_2_ catalysts were tested to assess plasma‐catalyst synergistic effects. Two sets of experiments were conducted: (i) power‐variation tests (13, 17, and 21 W) at a fixed total flow rate of 40 mL min^−1^, and (ii) flow‐rate‐variation tests (30–144 mL min^−1^, corresponding to SEI values of 40, 28.2, 16, and 8.3 kJ L^−1^) at a fixed discharge power of 20 W. Ammonia concentrations were analyzed online using the same GC system and calibration protocol.

Long‐term stability tests were performed using the double‐helix reactor packed with Ni/Al_2_O_3_. A pulsed power supply (MPP04‐A10A‐30, Kurita Co., Japan) operated at 10 kV and 12 kHz was applied. The reactor was supplied with N_2_/H_2_ = 1:1 at 200 mL min^−1^ under atmospheric pressure. Ammonia concentration was recorded every 15 min over 50 h (200 data points total). The hourly averaged NH_3_ concentration was used to calculate the ammonia synthesis rate and energy efficiency.

### 2.4 Electric‐Field Simulation and Plasma Diagnostics

#### 2.4.1 Electric Field Simulation

Electric‐field distributions for different reactor architectures were simulated using ANSYS Maxwell 2023 R2. The simulation geometry matched the experimental reactors exactly. A static potential of 2 kV was applied to the high‐voltage electrode, while the ground electrode was fixed at 0 V. All external boundaries were electrically insulated. Quartz was assigned a relative permittivity of 3.8, electrodes were treated as perfect conductors, and the discharge gap was filled with air (relative permittivity of 1). A steady‐state electrostatic solver with adaptive mesh refinement was employed, particularly in electrode regions and the discharge gap. The convergence criterion was set to 1 × 10^–6^. Simulation outputs included electric potential maps, electric‐field intensity contours (Figure S1), and axial/radial line profiles (Table S1). These results help identify the strong electric‐field regions that determine the initial breakdown locations. These high‐field zones are crucial because the microdischarges initiated there can supply seed electrons to the surrounding low‐field regions, thereby facilitating the development of larger‐area discharges within the reactor. This interpretation is further supported by the experimental discharge images presented in the following section.

#### 2.4.2 OES Analysis and Electronic Temperature Fitting

To assess the relative electron population under different discharge configurations, the emission intensity ratio *R* = *I*_N2+_(425 nm)/*I*_N2*_(335 nm) was used as a qualitative diagnostic. The 425 nm line corresponds to the N_2_^+^ first negative system, while the 335 nm line belongs to the N_2_ second positive system. Both are sensitive to electron impact processes, being the indicators of electron energy distribution within plasmas. Electronic temperatures were determined by fitting the 296–384 nm emission spectra using Lifbase software (SRI International, USA) based on the N_2_ second positive system emission bands. Rotational (*T*_rot_) and vibrational (*T*_vib_) temperatures were extracted from the fitted spectra. Prior to fitting, all spectra were baseline‐corrected and normalized to eliminate instrumental intensity variations. The obtained temperatures were used to characterize molecular excitation levels and electron energy distributions in plasmas. All OES measurements were conducted under the same conditions as the ammonia synthesis experiments: applied voltage 6 kV, discharge frequency 7 kHz, N_2_/H_2_ ratio 1:1, total flow 40 mL min^−1^, and atmospheric pressure.

## 3 Results and Discussion

### 3.1 Comparison of Ammonia Synthesis Performance

#### 3.1.1 Plasma‐Only Ammonia Synthesis

**FIGURE 2:**
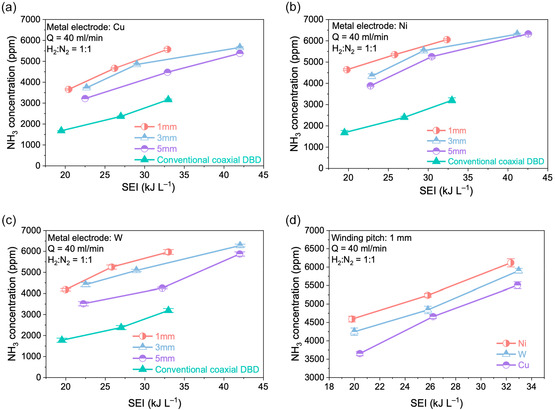
Comparison of plasma‐only ammonia synthesis performance under different electrode materials and winding spacings in the double‐helix DBD reactor. (a–c) NH_3_ concentration as a function of SEI for Cu, Ni, and W electrodes, respectively, at winding spacings of 1, 3, and 5 mm. The performance of the conventional coaxial DBD reactor is included for comparison. (d) Influence of electrode material at a fixed winding pitch of 1 mm. (Experiment conditions: total gas flow rate = 40 mL min^−1^; H_2_/N_2_ = 1:1 (v/v); atmospheric pressure; sine waveform input; discharge frequency = 7 kHz; applied voltage = 6–10 kV).

The double‐helix DBD reactor exhibited substantially enhanced ammonia synthesis performance compared with the conventional coaxial DBD configuration. Among all evaluated conditions, the Ni electrode with a 1 mm winding pitch delivered the highest NH_3_ concentration, achieving nearly a two‐fold increase relative to the conventional reactor at similar SEI values. As shown in Figure 2a–c, NH_3_ concentration increased monotonically with SEI across all electrode materials (Cu, Ni, and W), confirming that the ammonia formation rate is strongly correlated with the input energy density. At fixed SEI, reducing the winding pitch from 5 to 1 mm consistently enhanced NH_3_ production by approximately 30%, indicating that tighter electrode spacing promotes a stronger and more spatially uniform electric field, thereby increasing electron density and improving discharge efficiency, which promoted ammonia synthesis. Across all electrode types and pitch values, the double‐helix DBD reactor significantly outperformed the conventional coaxial DBD. The enhancement ranged from 100% to 202%, demonstrating that the symmetric dual‐dielectric configuration substantially improves discharge utilization and energy coupling into reactive species generation. Figure 2d compares the influence of electrode material under identical winding pitch (1 mm). With the discharge gap and applied voltage held constant, the NH_3_ formation rate followed the order of Ni > Cu > W. The Ni electrode yielded a maximum NH_3_ concentration of 6392 ppm at SEI = 41.1 kJ L^−1^, which is approximately 22% higher than Cu and 10% higher than W. This ranking underscores the significant impact of electrode material on plasma characteristics. Ni provides more efficient electron emission and superior energy transfer to the gas phase, thereby enhancing vibrational and electronic excitation of N_2_ and facilitating its dissociation pathways. These findings highlight that electrode material, winding pitch, and reactor architecture collectively govern the efficiency of plasma‐driven ammonia synthesis. The superior performance of the double‐helix configuration confirms that optimizing the spatial electric‐field distribution is critical for maximizing discharge activity and enhancing ammonia yield.

#### 3.1.2 Plasma‐Catalytic Ammonia Synthesis

Incorporating catalysts into the double‐helix DBD reactor significantly enhanced ammonia synthesis performance, with Ni/Al_2_O_3_ consistently outperforming Ni/ZrO_2_ and plasma‐only systems. Compared with the catalyst‐free system, Ni/Al_2_O_3_ improves NH_3_ concentration by up to 89% and enhances energy efficiency by up to 120%, highlighting the strong plasma‐catalyst synergy in the system under investigation. Catalyst characterization provides insight into the physicochemical properties of Ni/Al2O_3_. XRD analysis (Figure S4a) shows broader and weaker Ni diffraction peaks for Ni/Al_2_O_3_ relative to Ni/ZrO_2_, indicating smaller Ni crystallites (≈33 nm vs. 64 nm) and thus higher metal dispersion. H_2_‐TPD profiles (Figure S4b) further show a significantly larger desorption area for Ni/Al_2_O_3_, suggesting enhanced hydrogen activation and spillover capability. Finally, NH_3_‐TPD and N_2_‐TPD patterns (Figures S4c,d) reveal more intense desorption peaks in the 211°C–589°C range, demonstrating a higher density of accessible acid–base sites that facilitate N_2_ adsorption and NH_3_ intermediate stabilization*.* TEM analysis of the catalyst microstructures (Figure S5b,d) reveals that metallic Ni is highly uniformly dispersed on the surface of the Al_2_O_3_ support in the Ni/Al_2_O_3_ catalyst. The N_2_ adsorption–desorption isotherms of Ni/Al_2_O_3_ and Ni/ZrO_2_ are similar (Figure S6), both exhibiting type IV isotherms, indicating the presence of mesoporous structures in both catalysts. The Ni/Al_2_O_3_ catalyst exhibits a BET‐specific surface area of 156 m^2^ g^−1^ and a pore volume of 0.8 cm^3^ g^−1^, which are substantially higher than those of Ni/ZrO_2_ (Table S2; 21 m^2^ g^−1^; pore volume: 0.2 cm^3^ g^−1^). These structural and surface properties collectively indicate that Ni/Al_2_O_3_ provides more active sites and stronger H_2_ activation, enabling superior catalytic performance under plasma conditions.

**FIGURE 3:**
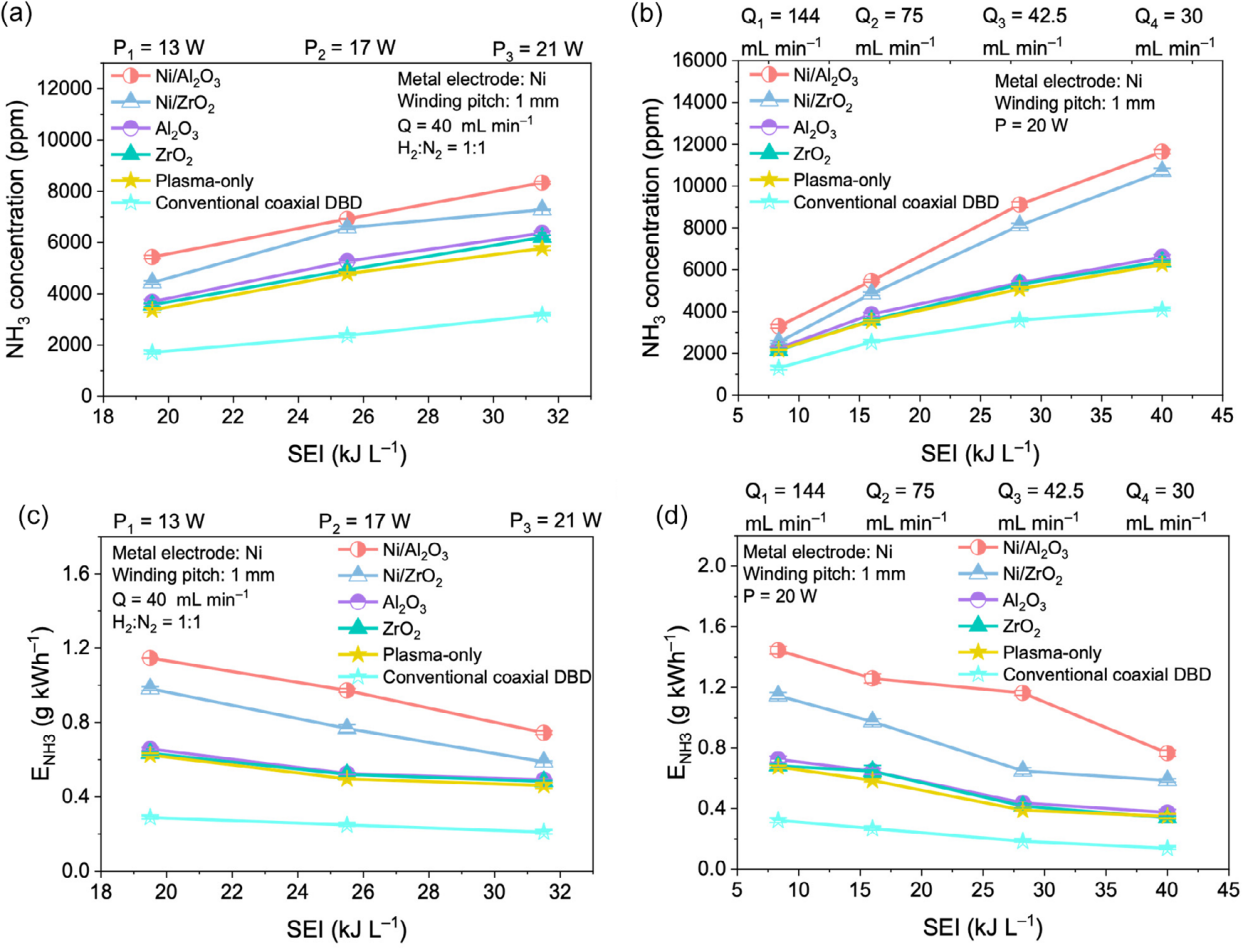
Ammonia synthesis performance of the double‐helix DBD reactor with and without catalyst incorporation: (a) NH_3_ concentration as a function of SEI at different input powers; (b) corresponding energy yield (*E*_NH3_) under the same conditions of (a); (c) NH_3_ concentration as a function of SEI at different gas flow rates under fixed discharge power (20 W); (d) corresponding *E*_NH3_ for the conditions shown in (c). Results from the plasma‐only and conventional coaxial DBD reactors are included for comparison. (Experiment conditions: total gas flow rate = 40 mL min^−1^; H_2_/N_2_ = 1:1 (v/v); atmospheric pressure; sine waveform input; discharge frequency = 7 kHz; applied voltage = 6–10 kV).

As shown in Figure 3a,b, catalyst incorporation in plasma discharge improves ammonia synthesis efficiency, while the double‐helix DBD reactor consistently outperforms the conventional coaxial DBD configuration under all tested conditions. Before evaluating catalytic performance, the blank supports (Al_2_O_3_ and ZrO_2_) were tested and showed negligible NH_3_ formation across the entire SEI range, confirming that the support materials themselves do not contribute to ammonia synthesis and do not influence the overall reaction trend. At a fixed flow rate of 40 mL min^−1^, NH_3_ concentration increased steadily with SEI for all systems. Ni/Al_2_O_3_ consistently delivers the highest NH_3_ yield across the entire SEI range, reaching 11,544 ppm at SEI = 40 kJ L^−1^, approximately 7% higher than Ni/ZrO_2_ and 1.85 times that of the plasma‐only system. Ni/ZrO_2_ shows moderate improvement over the catalyst‐free system, though its stronger Ni–support interaction and limited spillover restrict overall activity. The corresponding *E*_NH3_ (Figure 3c,d) followed the same performance hierarchy. Although *E*_NH3_ naturally decreases with increasing SEI, reflecting reduced energy utilization at higher power densities, Ni/Al_2_O_3_ retains the highest energy efficiency, achieving 1.44 g NH_3_ kWh^−1^ at SEI = 8.32 kJ L^−1^. This value surpasses Ni/ZrO_2_ by 27% and the plasma‐only reactor by 2.2‐fold, suggesting stronger electron–surface coupling and higher utilization of reactive species provided by the Ni/Al_2_O_3_ interface.

**FIGURE 4:**
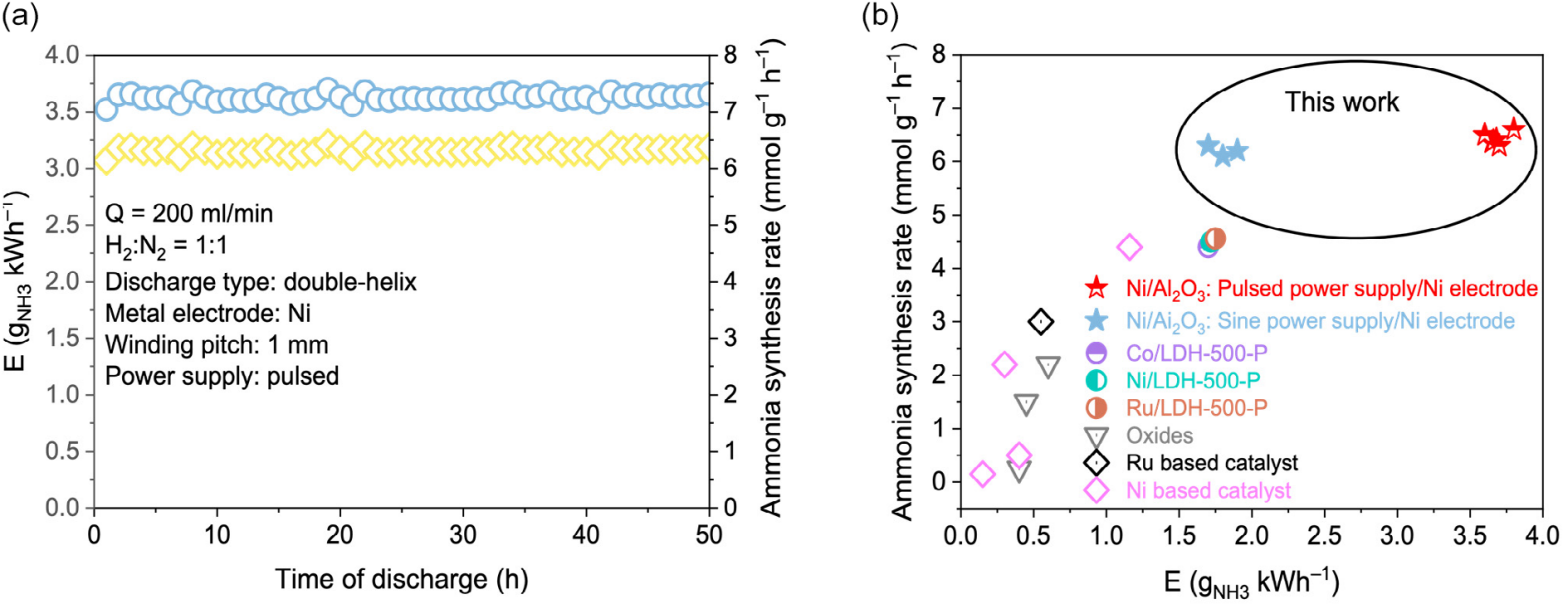
(a) Long‐term ammonia synthesis performance of the double‐helix DBD reactor coupled with the Ni/Al_2_O_3_ catalyst under pulsed power excitation, showing stable energy efficiency and ammonia synthesis rate over 50 h of continuous operation (experiment conditions: total gas flow rate = 150 mL min^−1^ with H_2_/N_2_ = 1:1 (v/v); atmospheric pressure; pulsed waveform input; discharge frequency: 12 kHz; applied voltage: 10 kV). (b) Benchmark comparison of ammonia synthesis rate and energy efficiency with representative plasma‐assisted systems reported in the literature.

Under a fixed discharge power of 20 W and varying gas flow rates (*Q* = 144–30 mL min^−1^; Figure 3c,d), the same activity trend was maintained, i.e., Ni/Al_2_O_3_ > Ni/ZrO_2_ > plasma‐only > conventional coaxial DBD. NH_3_ concentration increases with SEI as the flow rate decreases, primarily due to longer residence time and enhanced electron–molecule collision probability. Even at higher flow rates (*Q* = 600 mL min^−1^, SEI = 2 kJ L^−1^; Figure 4), Ni/Al_2_O_3_ sustains stable NH_3_ production without deactivation or discharge fluctuation, demonstrating its robustness. Based on XRD, N_2_‐TPD, and NH_3_‐TPD analyses, Ni/Al_2_O_3_ exhibits more favorable metal structures, stronger H_2_ activation capability, and a higher density of acid–base sites, which collectively enhance plasma–catalyst coupling and enable superior NH_3_ synthesis performance compared with Ni/ZrO_2_ and the plasma‐only system.

#### 3.1.3 Long‐Term Stability and Performance Benchmarking

The long‐term stability of the double‐helix DBD reactor coupled with the Ni/Al_2_O_3_ catalyst was evaluated under pulsed excitation (Figure 4a). The system exhibited excellent operational durability over 50 h, maintaining an energy efficiency of 3.68 g_NH3_ kWh^−1^ and a stable ammonia synthesis rate of 6.4 mmol g^−1^ h^−1^. This performance represents nearly a 90% improvement in energy efficiency compared with operation under the input of a sine waveform. The enhanced performance can be attributed to the unique characteristics of pulsed power discharge. Nanosecond‐scale voltage pulses generate extremely high instantaneous electric fields, enabling electrons (owing to their light mass) to rapidly acquire kinetic energy in the range of 1–10 eV. In contrast, ions and neutral species, which are several orders of magnitude heavier, respond sluggishly due to inertial limitations [20]. This selective acceleration allows electrons to excite molecules through collisions while maintaining low gas temperature, thereby reducing energy consumption and suppressing side reactions. Consequently, plasma‐catalytic efficiency is further enhanced under pulsed excitation, leading to higher ammonia production rates and higher energy efficiency.

Figure 4b benchmarks the performance of the present system against representative plasma‐catalytic ammonia synthesis studies reported in the literature. The Ni/Al_2_O_3_ catalyst tested in the double‐helix reactor clearly surpasses conventional oxides [21, 22, 23], and Ni‐based [22, 23, 24, 25, 26] and Ru‐based [27] catalysts in combined energy efficiency and ammonia synthesis rate. Using the Sumitomo power supply, the system achieved an energy efficiency of approximately 1.9 g NH_3_ kWh^−1^ with a corresponding formation rate of 6.2 mmol g^−1^ h^−1^, already comparable to or better than many state‐of‐the‐art Ni‐based and LDH‐derived catalysts. Under pulsed power operation, the energy efficiency further increased to 3.68 g NH_3_ kWh^−1^, placing this work among the top‐performing plasma‐catalytic ammonia synthesis systems to date. These results demonstrate that the synergistic combination of the double‐helix electrode architecture and pulsed power excitation leads to markedly improved plasma utilization and stronger plasma‐catalyst coupling. The reactor thus achieves an attractive balance between energy efficiency and productivity, underscoring its strong potential for next‐generation plasma‐assisted ammonia synthesis and distributed green ammonia technologies.

### 3.2 Diagnostic and Mechanistic Analysis of the Double‐Helix DBD Reactor

#### 3.2.1 Electric‐Field Simulation and Discharge Analysis

**FIGURE 5:**
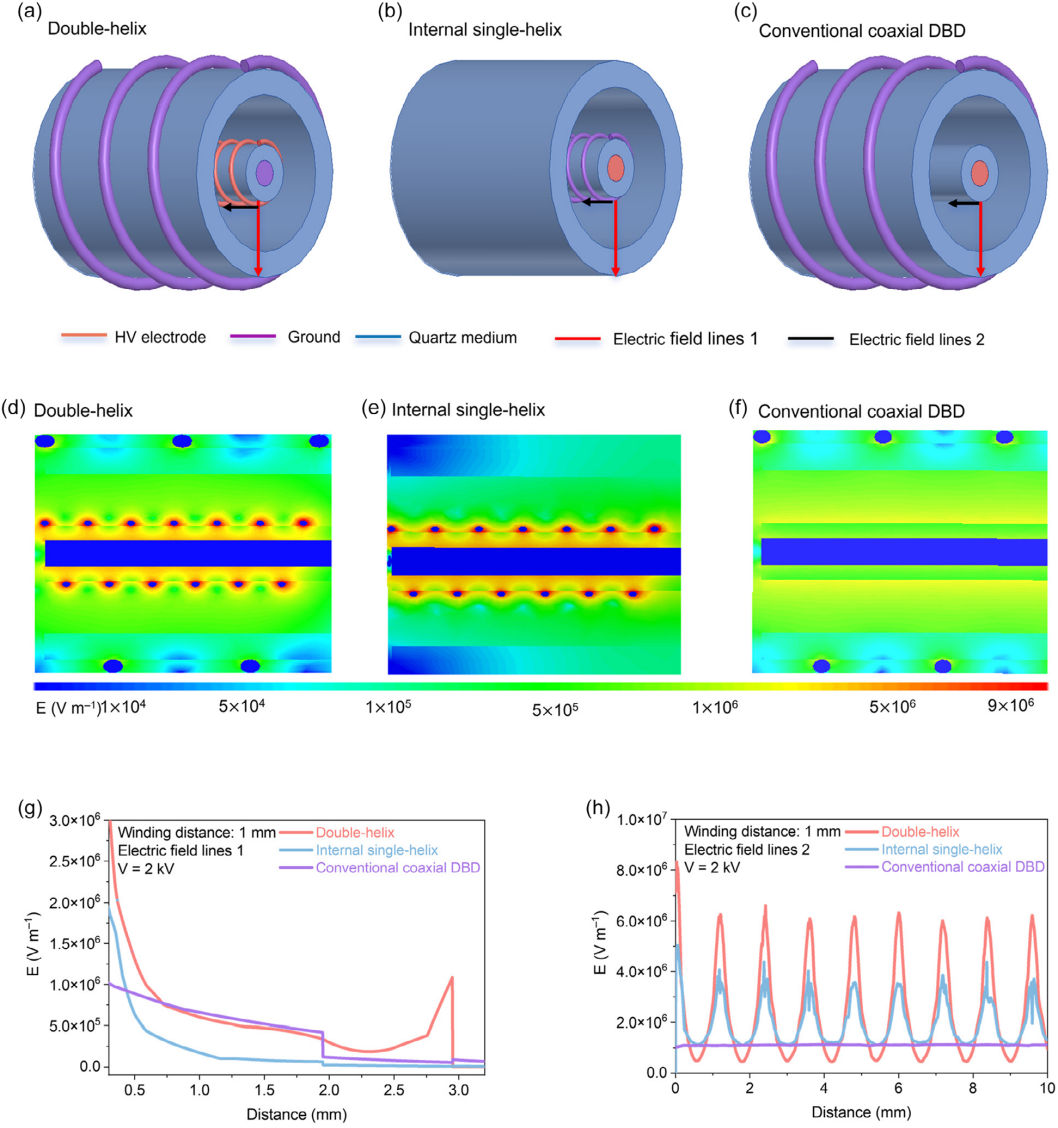
Electric‐field simulation of different DBD reactor architectures. (a–c) Schematic diagrams of the three electrode configurations: (a) double‐helix structure, (b) internal single‐helix structure, and (c) conventional coaxial DBD. (d–f) Simulated electric‐field intensity distributions (cross‐sectional contours) corresponding to the structures shown in (a–c). (g) Radial electric‐field intensity profiles (electric field line 1) for the three structures at an applied voltage of 2 kV and a winding pitch of 1 mm. (h) Axial electric‐field intensity profiles (electric field line 2) under the same conditions, illustrating the periodic field variation along the electrode winding direction.

Although electrostatic field simulations cannot describe the spatiotemporal evolution of the electric field after plasma ignition, previous studies have demonstrated that, prior to breakdown, quasistatic electrostatic models reliably capture geometry‐dependent field characteristics in corona and DBD reactors [28]. Analysis of the predischarge electric‐field distribution is therefore both appropriate and essential for identifying discharge initiation sites, comparing field intensities among different reactor geometries, and evaluating how electrode and dielectric configurations shape the spatial electric‐field pattern. On this basis, electrostatic simulations were employed to elucidate the physical origin of the enhanced ammonia synthesis performance observed in the double‐helix reactor by comparing the electric‐field strength and spatial distribution across three discharge configurations (Figure 5). The results clearly demonstrate that, under identical applied voltage, the double‐helix configuration generates both the strongest and the most uniformly distributed electric field across the discharge gap. The peak electric‐field strength reaches approximately 9 × 10^6^ V m^−1^, substantially exceeding that of the internal single‐helix structure (6 × 10^6^ V m^−1^) and the conventional coaxial DBD (1.5 × 10^6^ V m^−1^). This uniformly distributed, high‐intensity field establishes the physical foundation needed for stable volume discharge and efficient generation of reactive species.

Figure 5a–c depicts the electrode geometries, while Figure 5d–f illustrates their corresponding electric‐field cross sections. In the double‐helix configuration, the high‐voltage and ground electrodes are symmetrically positioned on opposite sides of the dual‐dielectric layers, producing a mirror‐symmetric potential distribution. This symmetry ensures that the electric field permeates continuously across the annular gap and concentrates at the gas‐dielectric interfaces, yielding a spatially uniform and intense field. Conversely, in the internal single‐helix design, the high‐voltage electrode is located adjacent to the inner tube wall, while the outer region lacks effective field confinement. As a result, the field intensity rapidly decays in the radial direction, and the strongest field appears near the electrode surface, suggesting that discharge initiation is restricted to a narrow region. The conventional coaxial DBD exhibits even stronger localization. In detail, high‐field zones are confined primarily to the outer dielectric surface, and the field strength within the gas gap is much weaker and decays sharply, consistent with surface‐dominated discharge behavior.

Quantitatively, the double‐helix DBD structure possesses an effective high‐field volume fraction of approximately 70%, which is roughly twice that of the internal single‐helix design (35%) and 3 times that of the conventional DBD (23%). This large high‐field volume strongly favors volumetric electron generation and stable, homogeneous microdischarge formation. These simulated trends are further corroborated by experimental observations, including discharge morphology and OES, as discussed below.

Figure 5g,h further compares the field‐strength profiles along two representative pathways. Along Line 1 (radial direction), the double‐helix electrode configuration exhibits the slowest attenuation of field strength, indicating a more persistent and stable radial field gradient. The order of radial attenuation follows: internal single‐helix > conventional DBD > double‐helix configuration. Along Line 2 (axial direction), which corresponds to the catalyst surface, the double‐helix configuration achieves a surface field strength of 8.2 × 10^6^ V m^−1^, surpassing the internal single‐helix (4.5 × 10^6^ V m^−1^) and the conventional DBD (1 × 10^6^ V m^−1^). The higher surface field intensity facilitates rapid electron acceleration and enhances electron‐impact excitation and dissociation of N_2_, promoting the formation of reactive intermediates such as N_2_* and N_2_^+^ [29].

The influence of winding spacing on the electric‐field morphology was also assessed for the double‐helix configuration (Figure S7). At a 1 mm winding pitch, the field lines are densely packed and uniformly distributed, indicating strong electrode coupling and continuous high‐field coverage throughout the gap. Increasing the pitch to 3 or 5 mm results in sparser field lines and contraction of the high‐field region toward the electrode surface, diminishing both field uniformity and effective discharge volume. These trends correlate directly with the experimental results in Figure 2a–c, where smaller pitches yielded higher NH_3_ concentration and superior energy efficiency.

Overall, the dual‐dielectric, symmetric geometry of the double‐helix configuration produces a stronger, more uniform electric field and significantly larger active discharge volume than the internal single‐helix or conventional coaxial structures. This enhanced field distribution increases electron energy density near the catalyst surface and broadens the active plasma reaction zone. The resulting gains in discharge uniformity and reactive species generation directly explain the experimentally observed improvements in ammonia synthesis (80%–120% enhancement vs. internal single‐helix and 2–3 times vs. plasma‐only, Figures 2–4), providing a robust physical basis for the superior performance of the double‐helix DBD reactor.

#### 3.2.2 Discharge Behavior Analysis via Morphological Observation and V–I Diagnostics

**FIGURE 6:**
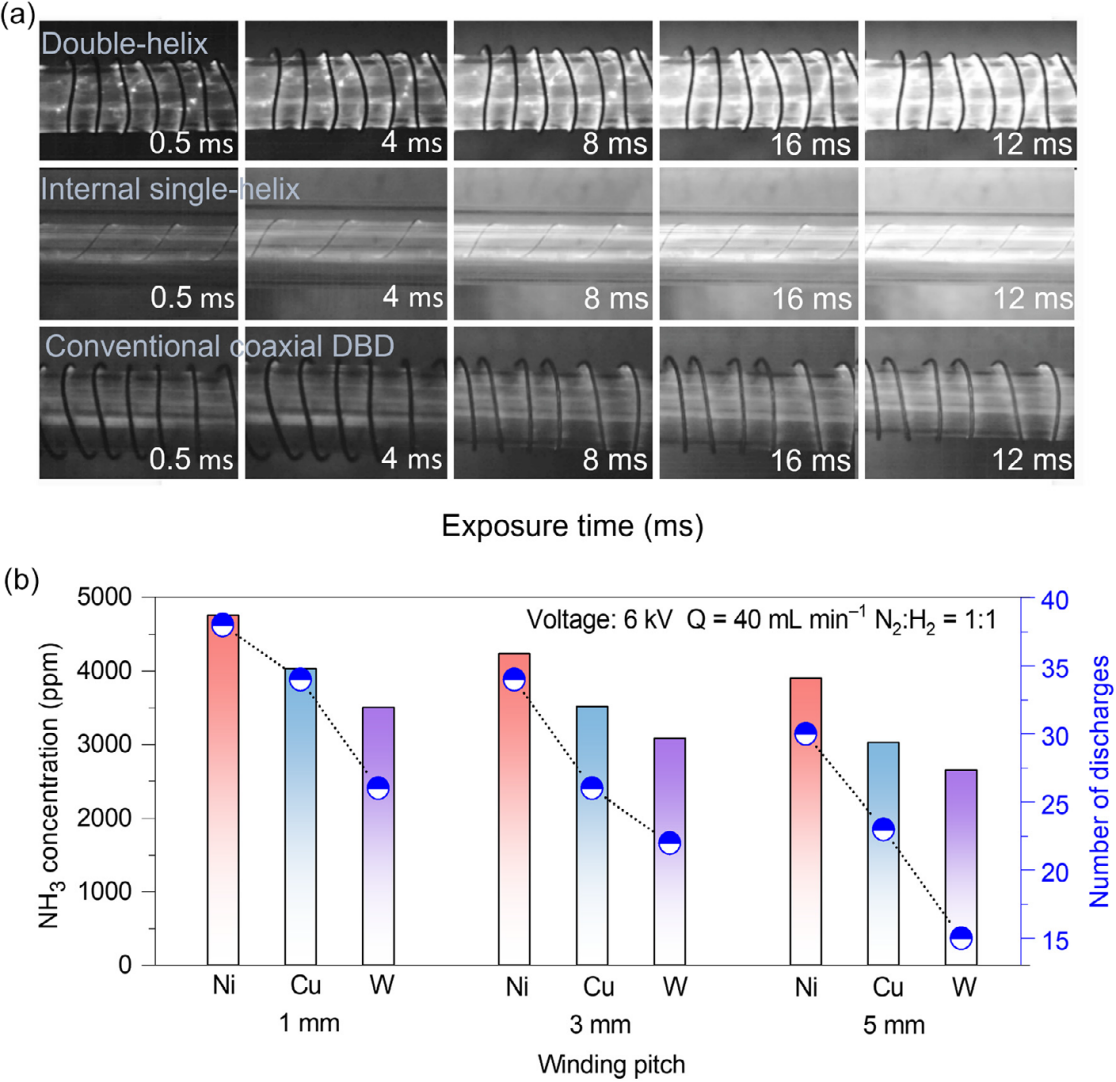
(a) High‐speed camera imaging of the instantaneous discharge evolution in the double‐helix, internal single‐helix, and conventional coaxial DBD reactors at different exposure times (0.5, 4, 8, 12, and 16 ms, under sine waveform input), illustrating differences in discharge initiation, propagation, and spatial uniformity. (b) Comparison of NH_3_ concentration (bars) and total discharge event counts per cycle (blue markers) for Ni, Cu, and W electrodes at different winding pitches (1, 3, and 5 mm). Discharge events were obtained from current signal statistics, where forward discharges were defined as ≥+44 mA and backward discharges as ≤−28 mA, and counted over one period (*T* = 142.8 μs). Experimental conditions (a): discharge electrode: Ni, winding pitch: 3 mm; (b): empty discharge zone; discharge voltage = 6 kV; discharge frequency = 7 kHz; total gas flow = 40 mL min^−1^; N_2_:H_2_ = 1:1; atmospheric pressure.

High‐speed imaging was employed to compare the spatiotemporal discharge evolution across the three reactor configurations under identical operating conditions (applied voltage: 6 kV; frequency: 7 kHz; total gas flow rate: 40 mL min^−1^; N_2_/H_2_ = 1:1). Figure 6a displays snapshots at exposure times of 0.5, 4, 8, 12, and 16 ms, revealing distinct differences in discharge formation and propagation among the reactors. At 0.5 ms, the double‐helix DBD reactor exhibits numerous bright spots and filamentary microdischarges distributed along the helical inner electrode, indicating a high probability of discharge initiation across the full annular gap. In contrast, the internal single‐helix structure shows discharge only in a small region adjacent to the inner electrode, while the conventional coaxial DBD displays sparse, surface‐limited microdischarges concentrated on the outer dielectric. At 4 ms, the luminous region in the double‐helix electrode configuration expands considerably, and microdischarge filaments begin merging into sheet‐like plasma zones, suggesting the early formation of a quasivolume discharge. Meanwhile, the internal single‐helix configuration maintains unilateral strip‐like discharges, and the conventional coaxial DBD remains confined to isolated surface filaments. By 8 ms, the discharge in the double‐helix configuration almost fills the entire annular gap, exhibiting a bright, continuous glow typical of a stable volume discharge. The internal single‐helix configuration becomes slightly brighter but still lacks uniformity, while the coaxial DBD continues to show localized surface discharges. At 12 ms, the double‐helix DBD reactor sustains a strong and spatially homogeneous glow, confirming robust discharge continuity. In contrast, both the internal single‐helix and conventional coaxial DBD configurations display alternating bright and dark zones, indicating intermittent, nonuniform discharge behavior dominated by surface modes. Finally, at 16 ms, the double‐helix configuration achieves a fully developed, spatially uniform volume discharge across the entire gap, whereas the internal single‐helix and conventional DBD reactors fail to establish complete volume discharge, exhibiting discontinuities and localized extinction. These results collectively show that the double‐helix architecture provides faster ignition, higher discharge density, and significantly improved spatial uniformity throughout the discharge cycle. These advantages arise from the internal single‐helix electrode in the double‐helix layout, which acts as a distributed predischarge site. Its localized, preferential microdischarges generate abundant seed electrons more uniformly within the annular gap [30], allowing earlier electron avalanche formation and supporting the rapid establishment of stable volume discharges. Consequently, the double‐helix DBD reactor achieves markedly enhanced discharge uniformity and energy utilization compared with the internal single‐helix and conventional coaxial DBD designs.

Figure 6b further quantifies the discharge characteristics by presenting discharge event statistics (Figures S8–S10) for Ni, Cu, and W electrodes at winding pitches of 1, 3, and 5 mm. Across all pitch values, the Ni electrode displays the highest number of discharge events within one period (*T* = 142.8 µs), reaching 38, 34, and 30 events at 1, 3, and 5 mm, respectively (Figure S8). On average, the discharge frequency for Ni is approximately 11% higher than for Cu (Figure S9) and 46% higher than for W (Figure S10), demonstrating that electrode material significantly influences discharge behavior. This trend may be rationalized by differences in relative magnetic permeability (*μ*_r_) among the three materials. Nickel possesses a much higher *μ*_r_ (∼600 H m^−1^) than copper or tungsten (∼1 H m^−1^). Under a pulse AC electric field, the Ni electrode concentrates magnetic flux more effectively, inducing a stronger transient electric field at the electrode surface [31, 32]. The elevated local electric field enhances electron acceleration and field emission, increasing the generation of seed electrons (via the *γ* effect, i.e., secondary electrons emitted from the electrode surface) and lowering the breakdown threshold. As a result, Ni electrodes facilitate more frequent microdischarge initiation under identical conditions.

**FIGURE 7:**
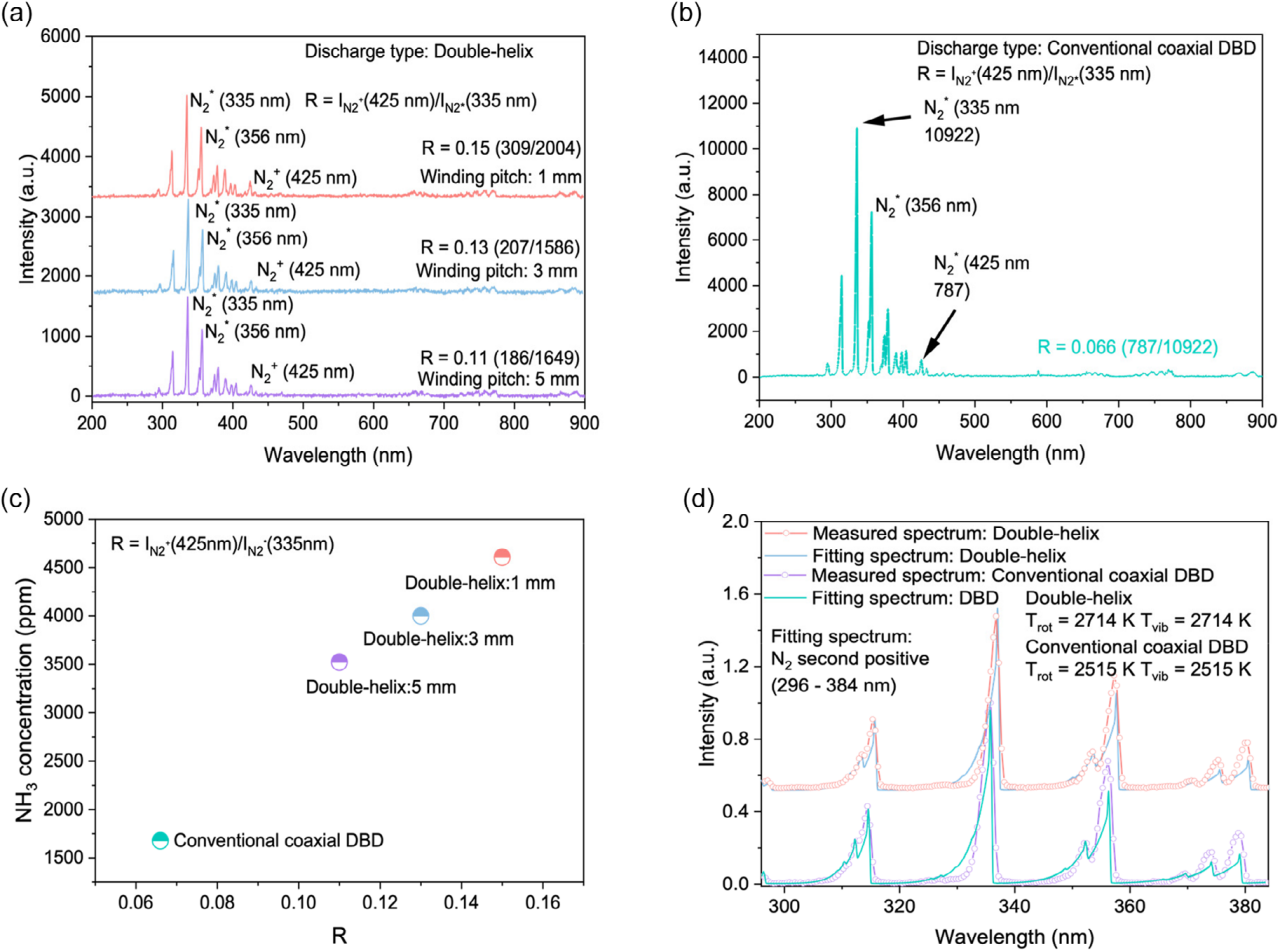
OES plasma parameter analysis under different discharge configurations and electrode winding pitches. (a) OES spectra of the double‐helix DBD reactor at winding pitches of 1, 3, and 5 mm; (b) OES spectrum of the conventional coaxial DBD reactor for comparison. (c) Correlation between the emission intensity ratio *R* = *I*_N2+_(425 nm)/*I*_N2*_(335 nm) and the corresponding NH_3_ concentration; (d) *T*_rot_ and *T*_vib_ temperatures extracted by fitting the N_2_ second positive system (294–384 nm) using Lifbase. Experimental conditions: Ni/Al_2_O_3_ catalyst; sine waveform input; discharge voltage = 6 kV; discharge frequency = 7 kHz; total flow rate = 40 mL min^−1^; H_2_:N_2_ = 1:1 (v/v); atmospheric pressure.

The increase in discharge events modifies the plasma characteristics in two key ways, i.e., higher electron density and energy, and expanded active plasma volume. Frequent microdischarges increase the average electron density and the fraction of energetic electrons, thereby intensifying electron–molecule inelastic collisions, which promotes N_2_ excitation and dissociation to form reactive nitrogen species (N_2_*, N_2_^+^, etc.), as corroborated by OES analysis (Figure 7). Because microdischarges occur at different spatial locations and times, they broaden the plasma coverage across the gap, reducing dark regions and promoting more uniform discharge (i.e., more effective plasma reaction volume). Together, these effects create a more reactive plasma environment when Ni is used as the high‐voltage electrode, explaining its superior ammonia synthesis performance (Figure 2), particularly at the optimal winding pitch of 1 mm.

### 3.3 Plasma Parameters Analysis and Temperature Fitting Results

OES was used to probe the electron energy characteristics of the different discharge configurations. OES results demonstrate that the double‐helix DBD reactor generates a significantly larger proportion of energetic electrons compared with the conventional coaxial DBD configuration. The ratio *R* = *I*_N2+_(425 nm)/*I*_N2*_(335 nm) was employed to quantitatively reflect the relative abundance of electrons in the discharge system [33]. As shown in Figure 7a, the double‐helix DBD reactor exhibits *R* values of 0.15, 0.13, and 0.11 at winding pitches of 1, 3, and 5 mm, respectively (Table S3), whereas the conventional coaxial DBD reactor shows an *R* value of only 0.066 (Figure 7b). Thus, depending on the winding pitch, the double‐helix configuration produces 127%, 96%, and 66% higher *R* values than the conventional system. These results indicate that, under identical applied voltage, the double‐helix configuration accelerates a greater fraction of electrons into high‐energy states, thereby exhibiting stronger molecular excitation and ionization capability. A clear positive correlation between the *R* value and ammonia concentration is observed (Figure 7c), demonstrating that a greater proportion of energetic electrons enhances ammonia formation. This trend is consistent with the discharge simulation (Figure S7a,b) and current pulse analysis results (Figure 5b), confirming that optimization of electric‐field distribution and generation of energetic electrons are key physical factors driving performance enhancement.

To further characterize molecular excitation, the rotational temperature (*T*_rot_) and vibrational temperature (*T*_vib_) of N_2_ were extracted from the fitted N_2_ second‐positive emission spectra [34], as shown in Figure 7d. The double‐helix configuration exhibits *T*_rot_ and *T*_vib_ ≈ 2714 K, higher than those of the conventional coaxial DBD (*T*_rot_ and *T*_vib_ ≈ 2515 K). This indicates a higher degree of molecular excitation and a larger population of electrons within the double‐helix configuration, which, in turn, facilitates more efficient excitation and ionization of N_2_ molecules. Taken together with electric‐field simulations (Figure 5) and discharge morphology/current analyses (Figure 6), the OES and temperature‐fitting results in Figure 7d provide a coherent plasma‐level explanation for the superior performance of the double‐helix DBD reactor. The symmetric dual‐dielectric layout generates a stronger and more spatially uniform electric field, promoting more efficient electron acceleration and increasing the fraction of electrons. This enhances the formation of reactive nitrogen species (N_2_*, N_2_^+^, etc.) and expands the active plasma reaction zone, consistent with the observed higher discharge frequency, stronger microdischarges, and greater plasma coverage. These synergistic plasma characteristics underpin the enhanced ammonia synthesis activity of the double‐helix DBD reactor compared with conventional DBD designs.

## 4 Conclusions

This work demonstrates that a double‐quartz‐tube coaxial double‐helix electrode DBD reactor provides a highly effective platform for plasma‐assisted ammonia synthesis. When coupled with Ni/Al_2_O_3_ catalyst, the plasma‐catalytic system achieves an energy yield of 3.68 g NH_3_ kWh^−1^ (pulsed excitation), placing it among the best‐performing plasma‐catalytic systems reported to date. The enhanced performance originates from the unique symmetric dual‐dielectric architecture, which fundamentally reshapes the spatial electric‐field distribution. Through integrated electric‐field simulations, optical emission spectroscopy, and high‐speed discharge imaging, we show that the double‐helix electrode configuration generates a significantly stronger and more uniform electric field than conventional DBD designs. This optimized field environment increases the density of electrons, promotes rapid avalanche development, and supports the transition from localized filamentary discharges to a stable, homogeneous volume‐discharge mode. These plasma characteristics directly enhance N_2_ activation, thereby amplifying the formation of reactive nitrogen species and improving the overall plasma‐catalyst synergy. Findings of this study highlight electric‐field engineering (via reactor‐architecture design) as a powerful and generic strategy for advancing nonthermal plasma‐assisted catalytic processes. The design principles demonstrated here offer clear potential for enabling low‐carbon, modular ammonia production and can be extended to other plasma‐enabled chemical transformations requiring efficient molecular activation under mild conditions.

## Supporting Information

Additional supporting information can be found online in the Supporting Information section. **Supporting** **Fig. S1**: Electric field distribution of three discharge configurations obtained from Maxwell simulation. (a–c) Internal single‐helix structure with winding pitches of 1, 3, and 5 mm, respectively; (d) Conventional coaxial DBD structure; (e–g) Double‐helix‐type structure with winding pitches of 1, 3, and 5 mm, respectively. **Supporting** **Fig. S2**: Schematic diagram of the experimental rig for plasma catalytic ammonia synthesis. **Supporting** **Fig. S3**: Gas chromatography calibration curve. **Supporting** **Fig. S4**: Characterization results of the Ni/Al_2_O_3_ and Ni/ZrO_2_ catalysts: (a) XRD patterns; (b) H_2_‐TPD profiles; (c) NH_3_‐TPD profiles; (d) N_2_‐TPD profiles. **Supporting** **Fig. S5**: TEM images of Ni/Al_2_O_3_ catalyst; (a) Distribution of Al particles under high‐magnification TEM; (b) Distribution of Ni particles under high‐magnification TEM; (c) Distribution of O particles under high‐magnification TEM; (d) Dispersion state of Ni particles on Al_2_O_3_ support. (e,f) TEM image of NI/Al_2_O_3__._ **Supporting** **Fig. S6**: BET measurements of Ni/Al_2_O_3_ and Ni/ZrO_2_ catalysts. **Supporting** **Fig. S7**: Comparison of electric field intensity along lines 1 and 2 among three structures under different winding pitches. (a) Field line 1 at 1 mm; (b) Field line 2 at 1 mm; (c) Field line 1 at 3 mm; (d) Field line 2 at 3 mm; (e) Field line 1 at 5 mm; (f) Field line 2 at 5 mm. **Supporting** **Fig. S8**: Discharge current waveforms of the Ni electrode at different winding pitches: (a) 1 mm; (b) 3 mm; (c) 5 mm. **Supporting** **Fig. S9**: Discharge current waveforms of the Cu electrode at different winding pitches: (a) 1 mm; (b) 3 mm; (c) 5 mm. **Supporting** **Fig. S10**: Discharge current waveforms of the W electrode at different winding pitches: (a) 1 mm; (b) 3 mm; (c) 5 mm. **Supporting** **Table 1**: Coordinate electric field lines 1 and 2 in simulation models for different winding pitches. **Supporting** **Table 2**: BET surface area and pore volume of Ni/Al_2_O_3_ and Ni/ZrO_2_ catalysts. **Supporting** **Table 3**: Comparison of OES spectra between the double‐helix‐type and conventional coaxial DBD structures. Definition: R=I_N2+_(425nm)/I_N2*_(335nm). Experimental conditions: V: 6 kV, Q = 40 mL min^−1^, f: 7 kHz.

## Author Contributions

**Shijie Xian**: data curation (lead), formal analysis (equal), investigation (lead), methodology (supporting), visualization (lead), writing – original draft (equal). **Xiaolan Fu**: data curation (supporting), formal analysis (equal), investigation (supporting), methodology (supporting), visualization (supporting), writing – original draft (equal). **Shaowei Chen**: data curation (supporting), formal analysis (supporting), investigation (supporting), methodology (supporting), supervision (supporting), writing – review and editing (supporting). **Liping Cao**: investigation (supporting), methodology (supporting), writing – review and editing (supporting). **Tianqi Liu**: funding acquisition (supporting), project administration (supporting), resources (supporting), writing – review and editing (supporting). **Yibing Mu**: investigation (supporting), methodology (supporting), project administration (lead), supervision (supporting), writing – review and editing (supporting). **Xiaolei Fan**: conceptualization (supporting), formal analysis (equal), funding acquisition (lead), methodology (supporting), supervision (equal), writing – review and editing (lead). **Jiangqi Niu**: conceptualization (lead), data curation (supporting), formal analysis (equal), investigation (supporting), methodology (lead), project administration (supporting), supervision (lead), writing – review and editing (supporting).

## Funding

This work was supported by the Innovative Research Group Project of the National Natural Science Foundation of China (W2431016); Leading Innovative and Entrepreneur Team Introduction Program of Zhejiang (2025R01022).

## Conflicts of Interest

The authors declare no conflicts of interest.

## Supporting information

Supplementary Material

## Data Availability

The data that support the findings of this study are available from the corresponding author upon reasonable request.
