# Supplementary material for: Improving Plasma‐Catalytic Ammonia Synthesis Using a Coaxial Double‐Helix‐Electrode Reactor
Source: ChemSusChem. 2026 Mar 29;19(7):e202502695. doi: 10.1002/cssc.202502695 (PMC13033345; doi:10.1002/cssc.202502695)
Supplement: Supplementary file 1 — Supplementary Material [file CSSC-19-e202502695-s001.pdf]

SUPPLEMENTARY INFORMATION

Improving Plasma Catalytic Ammonia Synthesis Using a Coaxial Double-Helix-Electrode Reactor

Table of Contents

Supplementary Tables
Supplementary Figures

Supplementary Tables

Table S1 Coordinate electric field lines 1 and 2 in simulation models for different winding pitches.

Electric field line coordinates	Start: (X , Y , Z)	End: (X , Y , Z)
Winding pitch 1mm line 1	1.16 , 0 , 1.7	4.5 , 0 , 1.7
Winding pitch 1mm line 2	1.16，0，0	1.16，0，10
Winding pitch 3mm line 1	1.16 , 0 , 4.8	4.5 , 0 , 4.8
Winding pitch 3mm line 2	1.16，0，0	1.16，0，10
Winding pitch 5mm line 1	1.16 , 0 , 7.7	4.5 , 0 , 7.7
Winding pitch 5mm line 2	1.16，0，0	1.16，0，10

Table S2 BET surface area and pore volume of Ni/Al_2O_3 and Ni/ZrO_2 catalysts

Catalyst Sample	BET surface area ($m^2\ g^{-1}$)	Pore volumes ($cm^3\ g^{-1}$)
Ni/Al_2O_3	156	0.8
Ni/ZrO_2	21	0.2

Table S3 Comparison of OES spectra between the double-helix-type and conventional coaxial DBD structures. Definition: $R=I_{N2+}(425nm)/I_{N2*}(335nm)$ Experimental conditions: V: 6 kV, Q = 40 mL min^{-1}, f: 7 kHz.

Winding pitch (mm)	R (double-helix)	R (conventional coaxial DBD)
1	0.15	
3	0.13	0.066
5	0.11	

Supplementary Figures

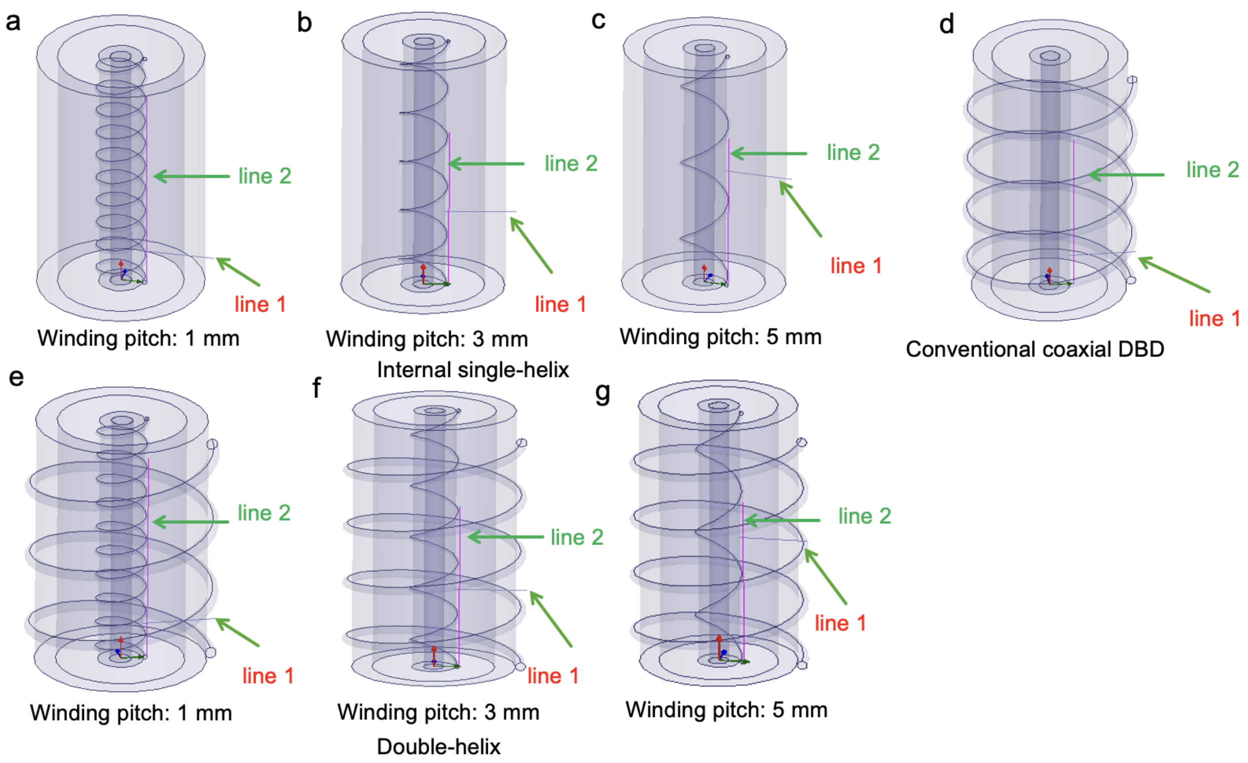

Figure S1. Electric field distribution of three discharge configurations obtained from Maxwell simulation. (a–c) Internal single-helix structure with winding pitches of 1, 3, and 5 mm, respectively; (d) Conventional coaxial DBD structure; (e–g) Double-helix-type structure with winding pitches of 1, 3, and 5 mm, respectively.

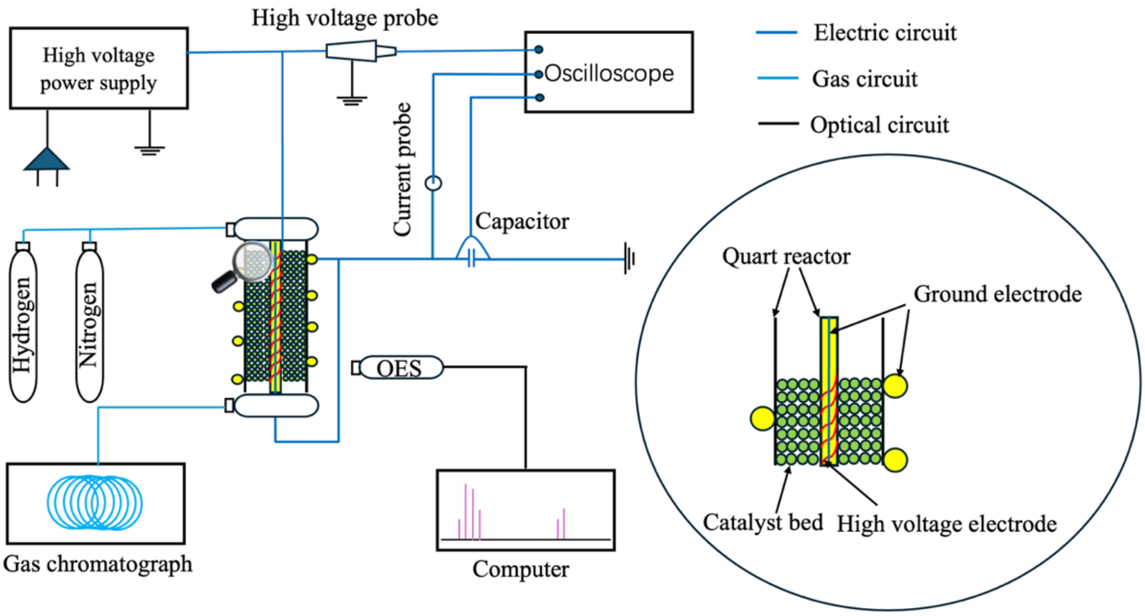

Figure S2. Schematic diagram of the experimental rig for plasma catalytic ammonia synthesis.

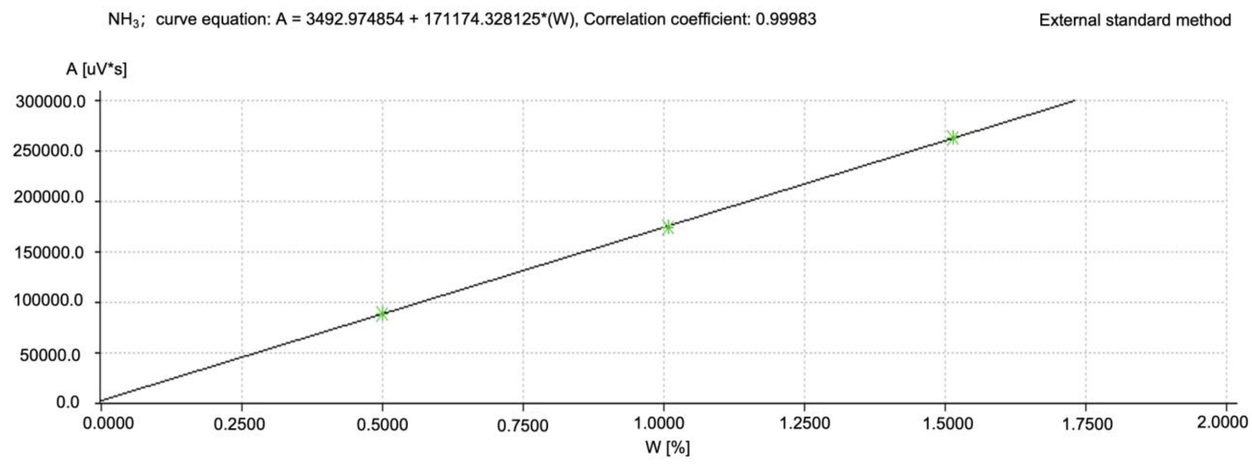

Figure S3. Gas chromatography calibration curve.

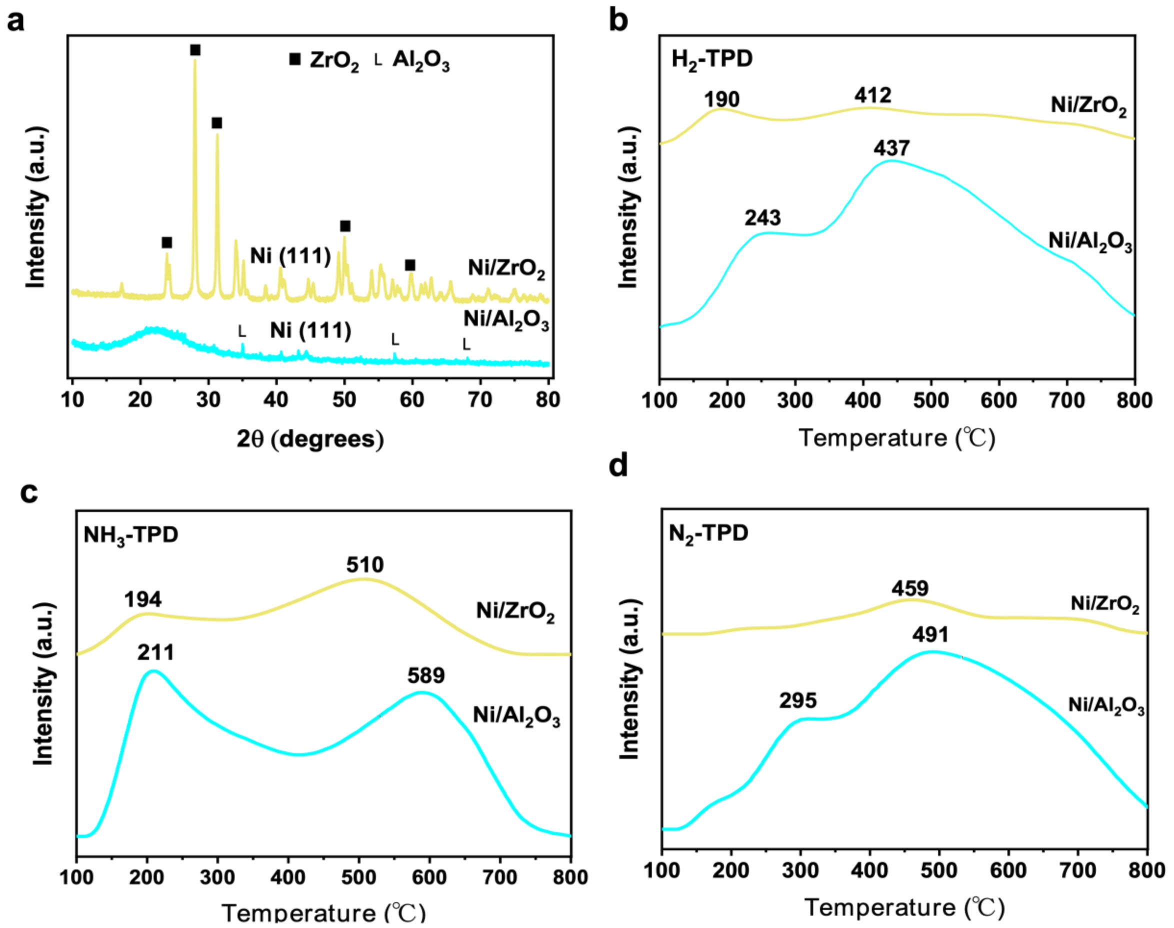

Figure S4. Characterization results of the Ni/Al_2O_3 and Ni/ZrO_2 catalysts: (a) XRD patterns; (b) H_2-TPD profiles; (c) NH_3-TPD. profiles; (d) N_2-TPD profiles.

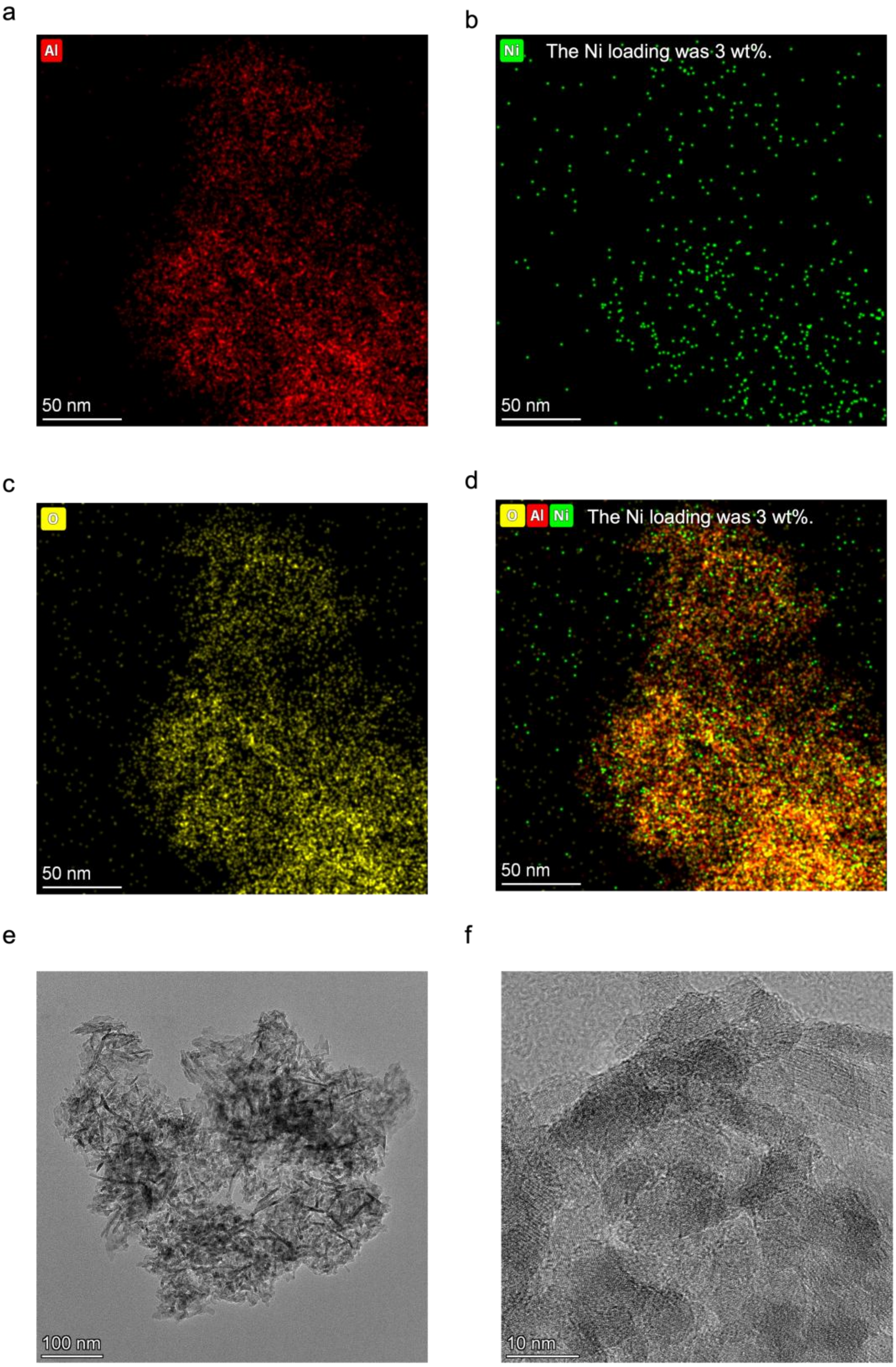

Figure S5. TEM images of Ni/Al_2O_3 catalyst; (a) Distribution of Al particles under high-magnification TEM; (b) Distribution of Ni particles under high-magnification TEM; (c) Distribution of O particles under high-magnification TEM; (d) Dispersion state of Ni particles on Al_2O_3 support. (e.f) TEM image of NI/Al_2O_3.

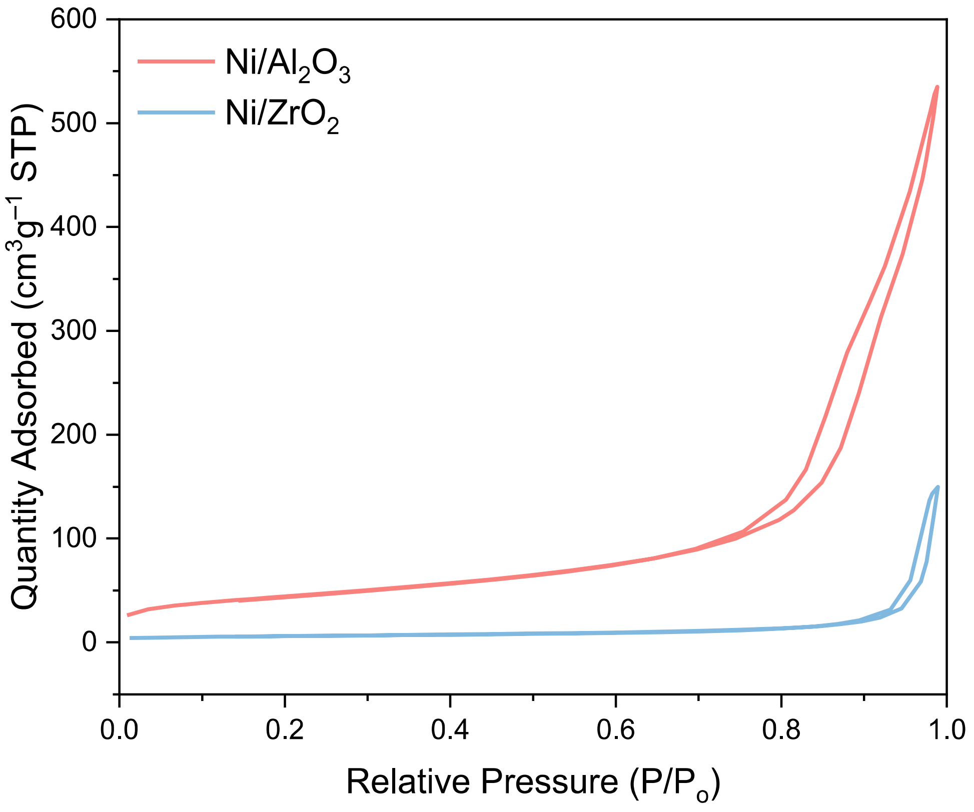

Figure S6. BET measurements of Ni/Al_2O_3 and Ni/ZrO_2 catalysts.

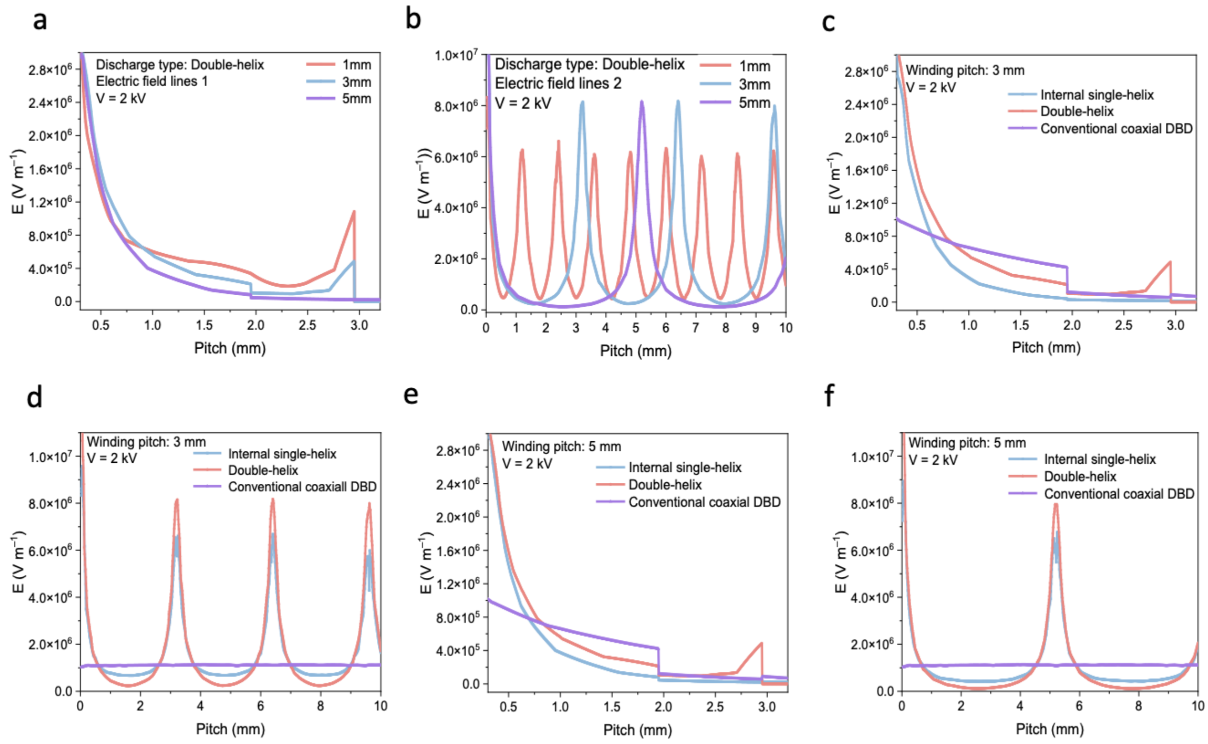

Figure S7. Comparison of electric field intensity along lines 1 and 2 among three structures under different winding pitches. (a) Field line 1 at 1 mm; (b) Field line 2 at 1 mm; (c) Field line 1 at 3 mm; (d) Field line 2 at 3 mm; (e) Field line 1 at 5 mm; (f) Field line 2 at 5 mm.

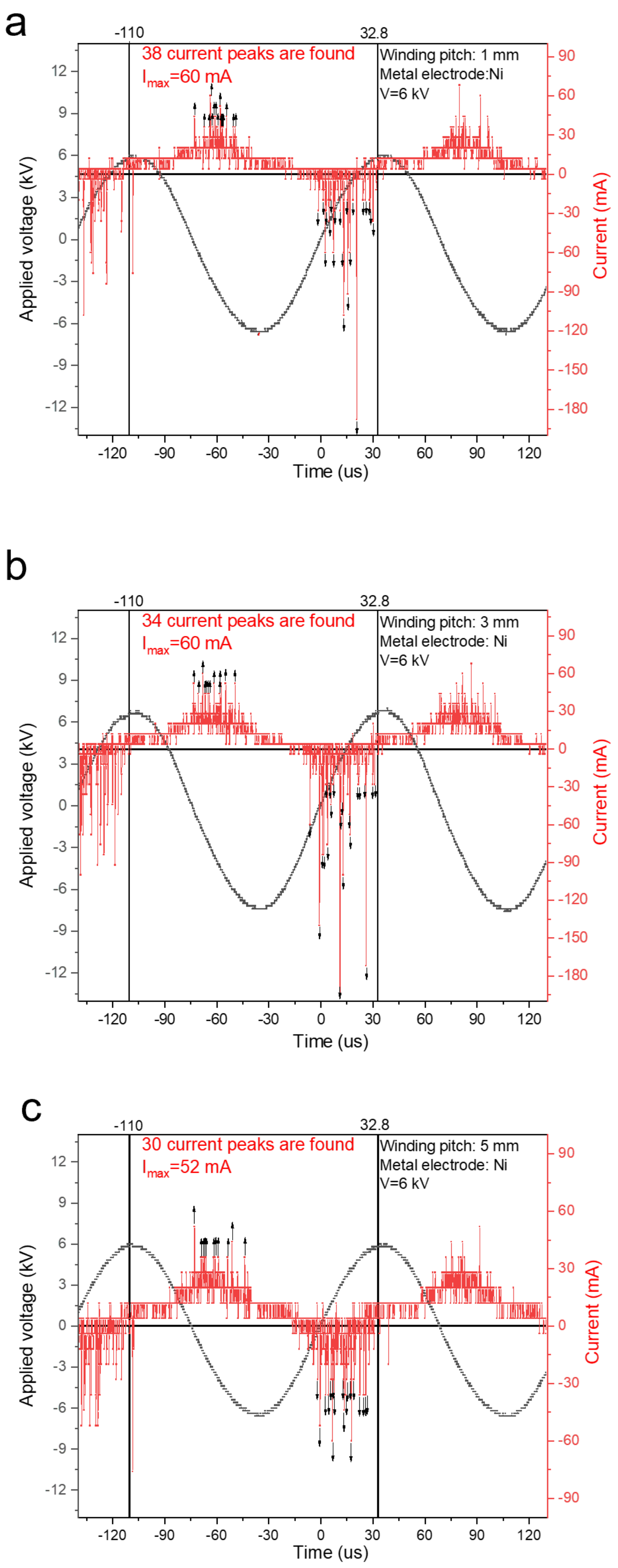

Figure S8. Discharge current waveforms of the Ni electrode at different d winding pitches: (a) 1 mm; (b) 3 mm; (c) 5 mm.

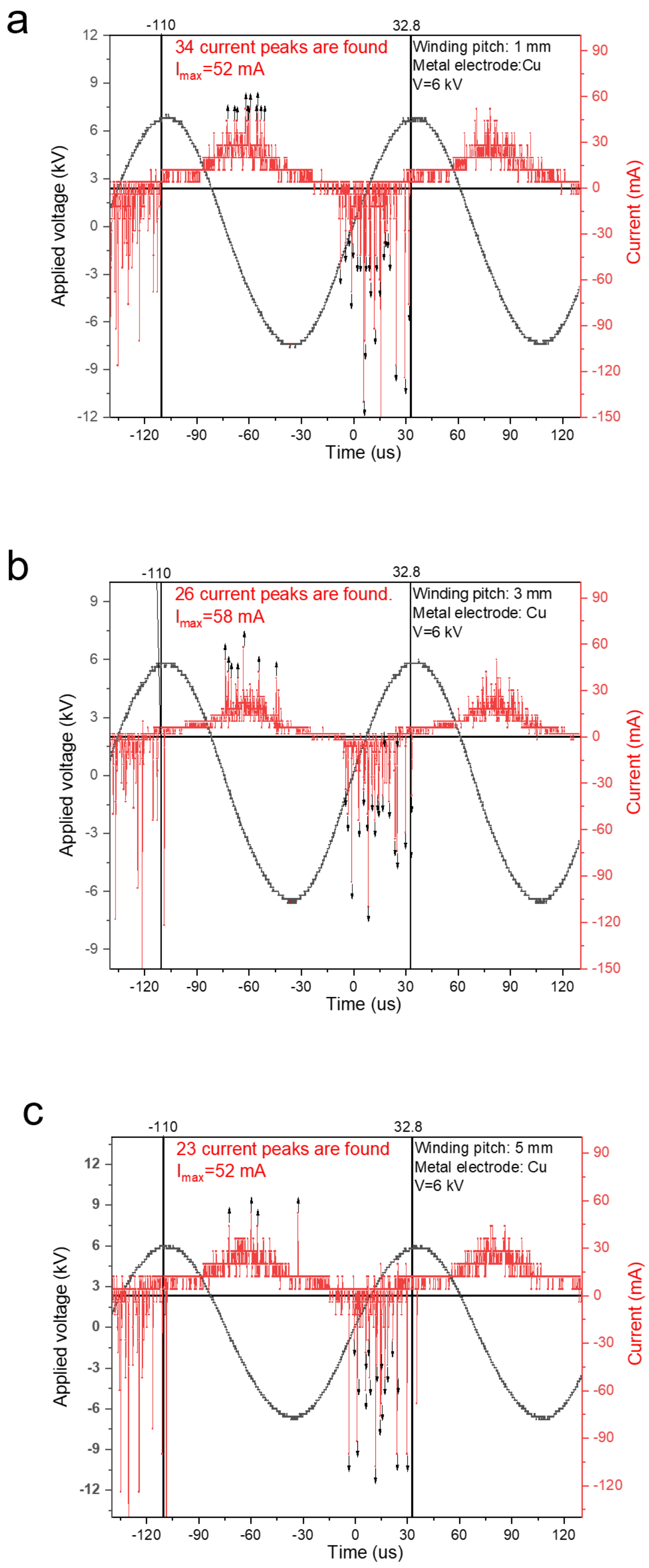

Figure S9. Discharge current waveforms of the Cu electrode at different d winding pitches: (a) 1 mm; (b) 3 mm; (c) 5 mm.

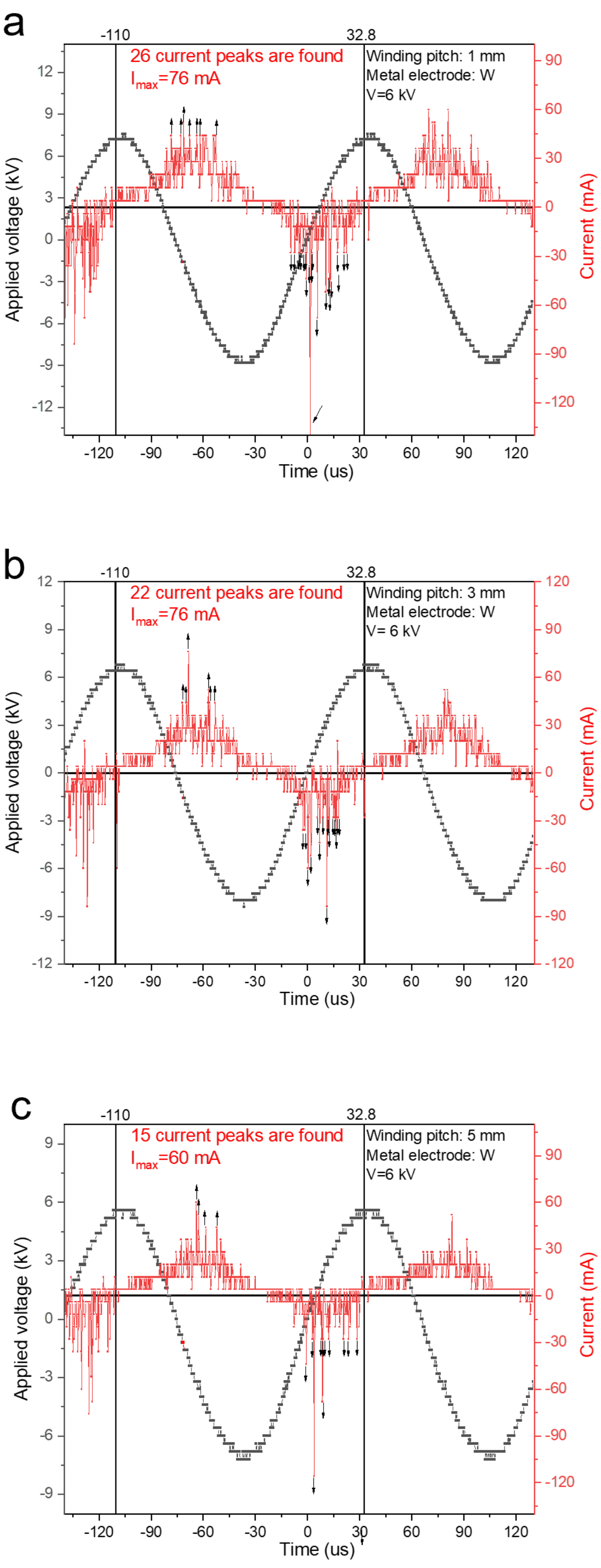

Figure S10. Discharge current waveforms of the W electrode at different d winding pitches: (a) 1 mm; (b) 3 mm; (c) 5 mm.
